# HGF and c-Met Interaction Promotes Migration in Human Chondrosarcoma Cells

**DOI:** 10.1371/journal.pone.0053974

**Published:** 2013-01-08

**Authors:** Hsi-Kai Tsou, Hsien-Te Chen, Ya-Huey Hung, Chia-Hao Chang, Te-Mao Li, Yi-Chin Fong, Chih-Hsin Tang

**Affiliations:** 1 Department of Neurosurgery, Taichung Veterans General Hospital, Taichung, Taiwan; 2 Department of Materials Science and Engineering, Feng Chia University, Taichung, Taiwan; 3 Department of Early Childhood Care and Education, Jen-Teh Junior College of Medicine, Nursing and Management, Miaoli County, Taiwan; 4 School of Chinese Medicine, College of Chinese Medicine, China Medical University, Taichung, Taiwan; 5 Department of Orthopedic Surgery, China Medical University Hospital, Taichung, Taiwan; 6 Graduate Institute of Basic Medical Science, China Medical University, Taichung, Taiwan; 7 Department of Orthopedic Surgery, Chang-Hwa Hospital, Department of Health, Executive Yuan, Chang-Hwa County, Taiwan; 8 Department of Pharmacology, School of Medicine, China Medical University, Taichung, Taiwan; University of Alabama at Birmingham, United States of America

## Abstract

Chondrosarcoma is a type of highly malignant tumor with a potent capacity for local invasion and causing distant metastasis. Chondrosarcoma shows a predilection for metastasis to the lungs. Hepatocyte growth factor (HGF) has been demonstrated to stimulate cancer proliferation, migration, and metastasis. However, the effect of HGF on migration activity of human chondrosarcoma cells is not well known. Here, we found that human chondrosarcoma tissues demonstrated significant expression of HGF, which was higher than that in normal cartilage. We also found that HGF increased the migration and expression of matrix metalloproteinase (MMP)-2 in human chondrosarcoma cells. c-Met inhibitor and siRNA reduced HGF-increased cell migration and MMP-2 expression. HGF treatment resulted in activation of the phosphatidylinositol 3′-kinase (PI3K)/Akt/PKCδ/NF-κB pathway, and HGF-induced expression of MMP-2 and cell migration was inhibited by specific inhibitors or siRNA-knockdown of PI3K, Akt, PKCδ, and NF-κB cascades. Taken together, our results indicated that HGF enhances migration of chondrosarcoma cells by increasing MMP-2 expression through the c-Met receptor/PI3K/Akt/PKCδ/NF-κB signal transduction pathway.

## Introduction

Hepatocyte growth factor (HGF) was identified in the early 1980s [1], [2] and was subsequently determined to be a heterodimeric molecule composed of an alpha and beta chain [3]. HGF has been reported to play critical roles in proliferation, migration, invasion, tumor angiogenesis, and lymphangiogenesis, recently [4], [5]. HGF transmitted the biological signal to target cells via an HGF receptor, the proto-oncogenic c-Met, which is a transmembrane tyrosine kinase receptor [6]. Recently, attention has increasingly been focused on c-Met because of its close association with and involvement in cancer [4]. Expression of HGF and c-Met has previously been detected in human cancer in abnormal stages, and is associated with a high tumor grade and poor prognosis [7], [8]. In addition, high circulating HGF levels are associated with lower survival and development of distant metastasis, and increases in circulating HGF are correlated with tumor size, nodal status, and histological evidence of venous invasion [9], [10]. These data suggest that HGF plays a critical role during cancer migration and metastasis.

Chondrosarcoma is the second most common primary malignant bone tumor after osteosarcoma and the commonest form of cancer in patients aged more than 20 years old. It has been found to be relatively resistant to radiotherapy and chemotherapeutic regimens [11]. Surgical resection remains the primary mode of therapy for chondrosarcoma. Since chondrosarcoma is a type of highly malignant tumor with a potent capacity for local invasion and distant metastasis [12], development of better strategies of treatment will ultimately require understanding of the molecular mechanisms of the steps involved during metastasis of human chondrosarcoma, and identification and specific targeting of the critical signaling effectors.

Metastasis involves multiple successive steps, including tumor adhesion in the primary site, invasion into the intravascular space, migration of tumor cells to distant sites, adhesion of tumor cells to vascular endothelium at distant sites, invasion into the surrounding tissues, and formation of secondary tumor colonies as a final step [13].

Matrix metalloproteinases (MMPs) are a family of more than 20 extracellular enzymes, which play important roles in the process of metastasis because their proteolytic activities assist in degradation of the extracellular matrix (ECM) and basement membrane [14], [15], [16]. It has been reported that cytokines, growth factors, chemokines, and MMPs regulate tumor cell migration and invasion through autocrine or paracrine pathways [17]. In human chondrosarcoma cell lines, MMP-1, MMP-2, MMP-3, MMP-9, and MMP-13 demonstrate increased expression [18]. Of these MMPs, MMP-2 has been reported to modulate the metastasis of human chondrosarcoma [19], [20]. Therefore, reduction of MMP-2 expression may be a good target for preventing or treating chondrosarcoma metastasis.

Recently studies have shown that HGF regulates cell migration and invasion in human cancer cells [21], [22]. However, the effect of HGF on migration activity in human chondrosarcoma cells is not well known. Here we show that HGF increases migration and up-regulates MMP-2 expression in human chondrosarcoma cells. Moreover, the c-Met receptor, phosphatidylinositol 3′-kinase (PI3K), Akt, protein kinase C (PKC) δ, and NF-κB signaling pathways were shown to be involved.

## Materials and Methods

### Materials

Anti-mouse and anti-rabbit IgG-conjugated horseradish peroxidase, rabbit polyclonal antibodies specific for β-actin, p85, p-p85, Akt, p-Akt, IKK, p-IKK, IκB, p-IκB, p65, p-p65, PKCδ, control shRNA, HGF shRNA, and siRNA against c-Met, PKCδ, c-Jun, SP-1, and scrambled control siRNA were purchased from Santa Cruz Biotechnology (Santa Cruz, CA, USA). Rottlerin, PDTC, and TPCK were purchased from Calbiochem (San Diego, CA, USA). Recombinant human HGF was purchased from R&D Systems (Minneapolis, MN, USA). Rabbit polyclonal antibody specific for PKCδ phosphorylated at Thr^505^ was purchased from Cell Signaling and Neuroscience (Danvers, MA, USA). The NF-κB luciferase plasmid was purchased from Stratagene (La Jolla, CA, USA). The p85α and Akt (Akt K179A) dominant-negative mutants were gifts from Dr. W.M. Fu (National Taiwan University, Taipei, Taiwan). The IKKα (KM) and IKKβ (KM) mutant plasmids were gifts from Dr. H. Nakano (Juntendo University, Tokyo, Japan). The pSV-β-galactosidase vector and luciferase assay kit were purchased from Promega (Madison, MA, USA). All other chemicals were purchased from Sigma–Aldrich (St. Louis, MO, USA).

### Cell Culture

The human chondrosarcoma cell line (JJ012) was kindly provided by the laboratory of Dr. Sean P. Scully (University of Miami School of Medicine, Miami, FL, USA) [23]. The human chondrosarcoma cell line (SW1353) was purchased from the American Type Culture Collection. Cells were cultured in Dulbecco’s modified Eagle’s medium/α-minimum essential medium supplemented with 10% fetal bovine serum and maintained at 37°C in a humidified atmosphere of 5% CO_2_.

### Migration Assay

The migration assay was performed using Transwell (Costar, NY, USA; pore size, 8-µm) in 24-well dishes. For the invasion assay, filters were precoated with 25 µl Matrigel basement membrane matrix (BD Biosciences, Bedford, MA, USA) for 30 min. The following procedures were the same for both migration and invasion assays. Before migration assay, cells were pretreated for 30 min with different concentrations of inhibitors, including the c-Met inhibitor, MMP-2 inhibitor, Ly294002, the Akt inhibitor, rottlerin, PDTC, TPCK, or vehicle control (0.1% DMSO). None of the inhibitors and siRNAs used in this study affected basal migration and MMP-2 expression (data not shown). Approximately 1×10^4^ cells in 200 µl of serum-free medium were placed in the upper chamber, and 300 µl of the same medium containing HGF was placed in the lower chamber; the inhibitors were retained after pretreatment. The plates were incubated for 24 h at 37°C in 5% CO_2_; then, cells were fixed in methanol for 15 min and stained with 0.05% crystal violet in PBS for 15 min. Cells on the upper side of the filters were removed with cotton-tipped swabs, and the filters were washed with PBS. Cells on the underside of the filters were examined and counted under a microscope. Each clone was plated in triplicate for each experiment, and each experiment was repeated at least 3 times. The number of invading cells in each experiment was adjusted by the cell viability assay to correct for proliferation effects of HGF treatment (corrected invading cell number = counted invading cell number/percentage of viable cells) [24], [25].

### Quantitative Real-time PCR

Total RNA was extracted from chondrosarcoma cell lines using a TRIzol kit (MDBio Inc., Taipei, Taiwan). The reverse transcription reaction was performed using 2 µg of total RNA that was reverse transcribed into cDNA using an oligo (dT) primer [26], [27]. Quantitative real-time PCR (qPCR) analysis was carried out using Taqman® one-step PCR Master Mix (Applied Biosystems, Foster City, CA). cDNA templates (2 µl) were added per 25-µl reaction with sequence-specific primers and Taqman® probes. β-actin was used as internal control. Sequences for all target gene primers and probes were purchased commercially (Applied Biosystems). qPCR assays were carried out in triplicate on a StepOnePlus sequence detection system. The cycling conditions involved a 10-min polymerase activation step at 95°C, followed by 40 cycles each consisting of 95°C for 15 s and 60°C for 60 s. The threshold was set above the non-template control background and within the linear phase of the target gene amplification to calculate the cycle number at which the transcript was detected (denoted C_T_).

### Western Blot Analysis

Cellular lysates were prepared as described previously [28], [29]. Proteins were resolved on SDS-PAGE and transferred to Immobilon polyvinyldifluoride membranes. The blots were blocked with 4% BSA for 1 h at room temperature and then probed with rabbit anti-human antibodies against PKCδ, p85, Akt, or p65 (1∶1000) for 1 h at room temperature. After 3 washes, the blots were subsequently incubated with donkey anti-rabbit peroxidase-conjugated secondary antibody (1∶1000) for 1 h at room temperature. The blots were visualized by enhanced chemiluminescence with Kodak X-OMAT LS film (Eastman Kodak, Rochester, NY).

### Zymography Analysis

The supernatants of JJ012 cells were mixed with sample buffer without reducing agent or heating. The samples were loaded into a gelatin (1 mg/mL)-containing SDS–polyacrylamide gel and underwent electrophoresis under constant voltage. Afterwards, the gel was washed with 2.5% Triton X-100 to remove SDS, rinsed with 50 mM Tris-HCl, pH 7.5, and then incubated overnight at room temperature with developing buffer (50 mM Tris-HCl, pH 7.5, 5 mM CaCl_2_, 1 µM ZnCl_2_, 0.02% thimerosal, 1% Triton X-100).

### Immunohistochemistry

A human chondrosarcoma tissue array was purchased from Biomax (Rockville, MD, USA). The tissue on glass slides were rehydrated and incubated in 3% hydrogen peroxide to block the endogenous peroxidase activity. After trypsinization, sections were blocked by incubation in 3% bovine serum albumin in PBS. The primary antibody monoclonal mouse anti-human HGF antibody was applied to the slides at a dilution of 1∶50 and incubated at 4°C overnight. After 3 washes in PBS, the samples were treated with goat anti-mouse IgG biotin-labeled secondary antibodies at a dilution of 1∶50. Bound antibodies were detected with an ABC kit (Vector Laboratories). The slides were stained with the chromogen diaminobenzidine, washed, counterstained with Delafield’s hematoxylin, dehydrated, treated with xylene, and mounted.

### Transfection and Reporter Gene Assay

Human chondrosarcoma cells were co-transfected with 0.8 µg κB-luciferase plasmid, 0.4 µg β-galactosidase expression vector. Cells were grown to 80% confluence in 12-well plates and were transfected on the following day using Lipofectamine 2000 (LF2000; Invitrogen, Carlsbad, CA, USA). DNA and LF2000 were premixed for 20 min and then applied to the cells. At 24 h after transfection, the cells were incubated with the indicated agents. After a further 24-h incubation, the media were removed, and cells were washed once with cold PBS. To prepare lysates, 100 µl reporter lysis buffer (Promega, Madison, WI, USA) was added to each well, and cells were scraped from dishes. The supernatant was collected after centrifugation at 13,000 rpm for 2 min. Aliquots of cell lysates (20 µl) containing equal amounts of protein (20–30 µg) were placed into the wells of an opaque black 96-well microplate. An equal volume of luciferase substrate was added to all samples, and luminescence was measured in a microplate luminometer. The value of luciferase activity was normalized to transfection efficiency, which was monitored by the co-transfected β-galactosidase expression vector.

### Chromatin Immunoprecipitation Assay

Chromatin immunoprecipitation (ChIP) analysis was performed as described previously [19]. DNA immunoprecipitated by anti-p65 antibody was purified. The DNA was then extracted with phenol-chloroform. The purified DNA pellet was subjected to PCR. PCR products were then resolved by 1.5% agarose gel electrophoresis and visualized by UV.

The primers: 5′-CCCCTGTTCAAGATGGAGTC-3′ and 5′-CCCAGGTTGCTTCCTTACCT-3′ were utilized to amplify across the human *MMP2* promoter region (−673 to −517).

### Establishment of Stably Transfected Cells

HGF shRNA or control shRNA plasmids were transfected into JJ012 cells using Lipofectamine 2000 transfection reagent. Twenty-four hours after transfection, stable transfectants were selected in puromycin (Life Technologies, Grand Island, NY, USA) at a concentration of 10 µg/mL. Thereafter, the selection medium was replaced every 3 days. After 2 weeks of selection in puromycin, clones of resistant cells were isolated.

### Wound-healing Migration Assay

For the wound-healing migration assay, cells were seeded in 12-well plates at a density of 1×10^5^ cells/well in culture medium. At 24 h after seeding, the confluent monolayer of cultured cells was scratched with a fine pipette tip, and migration was visualized by microscope under magnification. The rate of wound closure was observed at the indicated time.

### Patients and Specimen Preparation

The study protocol was approved by the Institutional Review Board of China Medical University Hospital, and all subjects gave informed written consent before enrollment. The specimens of tumor tissue, normal cartilage tissue, or normal bone were obtained from patients who had been diagnosed with chondrosarcoma or knee osteoarthritis and had undergone surgical resection at China Medical University Hospital. Tissue specimens were ground and then sonicated in TRIzol kit. mRNA levels were analyzed using qPCR analysis.

### Statistics

The values reported are means ± S.E. Statistical analyses between 2 sample groups were performed using Student’s *t-*test. Statistical comparisons of more than 2 groups were performed using one-way analysis of variance (ANOVA) with Bonferroni’s *post-hoc* test. In all cases, *p*<0.05 was considered significant.

## Results

### HGF Increases Migration in Human Chondrosarcoma

**Figure 1:**
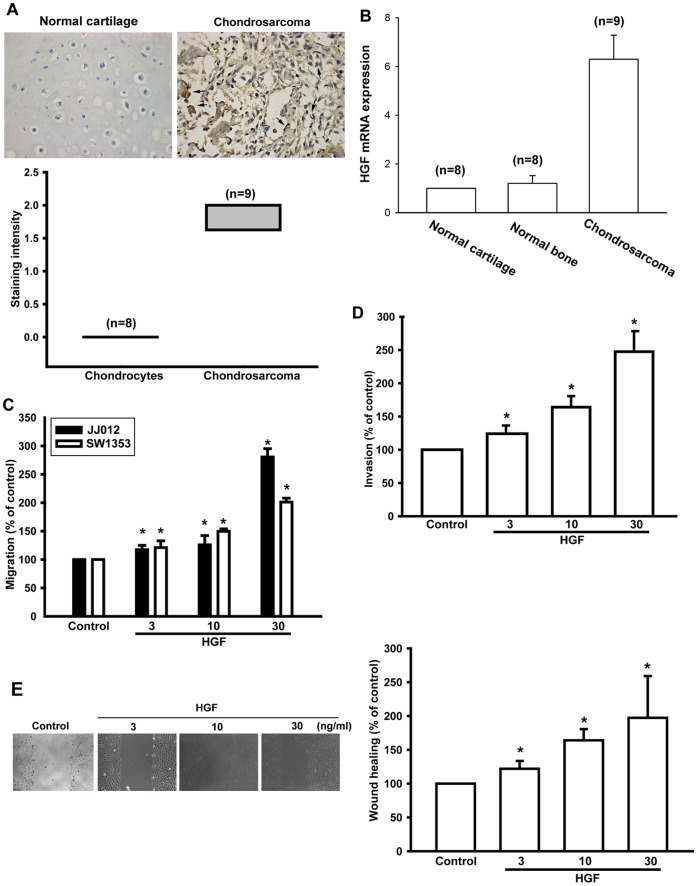
HGF induced the migration of human chondrosarcoma cells. (A) Immunohistochemistry showing HGF expression in normal cartilage and chondrosarcoma tissues (arrow shows HGF staining). Quantitative data are shown in the lower panel. (B) Quantitative PCR analysis for HGF mRNA expression in normal cartilage, normal bone, and chondrosarcoma tissues. (C and D) Cells were incubated with HGF (3–30 ng/mL), and *in vitro* migration and invasion was measured by Transwell assay after 24 h. (E) JJ012 cells were treated with HGF for 24 h, after which the wound-scratching assay was performed. Results are expressed as mean ± S.E. *p<0.05 compared with control; #*p*<0.05 compared with HGF-treated group.

HGF has been reported to promote directional migration and invasion of human cancer cells [21], [22]. However, little is known about the expression of HGF in human chondrosarcoma cells. As a first step, we examined human chondrosarcoma patients for expression of HGF using immunohistochemistry. We found that the expression of HGF in tissue from chondrosarcoma patients was significantly higher than in normal cartilage (Fig. 1A, arrow shows the HGF staining). The quantification data of HGF protein expression are shown in Fig. 1A lower panel. In addition, the qPCR results also showed that the HGF mRNA expression levels in chondrosarcoma patients were higher than in normal cartilage as well as in normal bone (Fig. 1B). To elucidate a link between HGF expression and chondrosarcoma cell migration, we next examined the migratory activity of human chondrosarcoma by using the Transwell assay. Stimulation of human chondrosarcoma cells (JJ012 and SW1353 cells) with HGF increased migration activity dose-dependently (Fig. 1C). We also found that HGF increased the invasive ability of JJ012 cells through a Matrigel basement membrane matrix (Fig. 1D). Furthermore, the wound-scratching assay demonstrated that HGF increased wound healing activity in human chondrosarcoma cells (Fig. 1E). Thus, expression of HGF was associated with an invasive and/or metastatic phenotype of chondrosarcoma cells.

### Involvement of MMP-2 in HGF-directed Migration of Chondrosarcoma Cells

**Figure 2:**
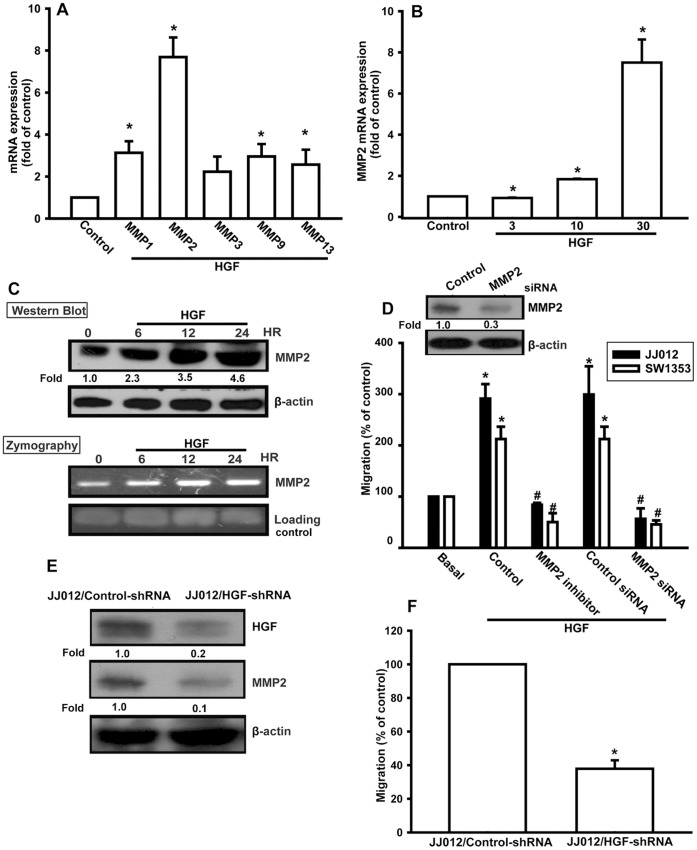
HGF-directed migration activity of human chondrosarcoma cells involves up-regulation of MMP-2. (A) JJ012 cells were incubated with HGF (30 ng/mL) for 24 h, the mRNA levels of MMP-1, −2, −3, −9, and −13 were determined using qPCR. (B) JJ012 cells were incubated with HGF for 24 h, and the mRNA level of MMP-2 then determined using qPCR. (C) JJ012 cells were incubated with HGF for the indicated time intervals. The cultured medium and cell lysates were then collected. The protein level of MMP-2 in cell lysates and the enzyme activity of MMP-2 in supernatants were examined by Western blotting and zymography. (D) JJ012 cells were treated with MMP-2 inhibitor for 30 min or were transfected with MMP-2 siRNA for 24 h, followed by stimulation with HGF; *in vitro* migration was then measured after 24 h. (E) Protein levels of HGF and MMP-2 in JJ012/control-shRNA and JJ012/HGF-shRNA cells was examined by western blotting. (F) I*n vitro* migration activity of JJ012/control-shRNA and JJ012/HGF-shRNA cells was measured using a Transwell assay. Results are expressed as mean ± S.E. *p<0.05 compared with control; #*p*<0.05 compared with HGF-treated group.

It has been reported that human chondrosarcoma cells significantly express MMP-1, −2, −3, −9, and −13 [18]. We therefore, hypothesized that any of these MMPs may be involved in HGF-directed chondrosarcoma cell migration. Incubation of cells with HGF induced the expression of MMP-2, as seen using qPCR (Fig. 2A). On the other hand, HGF also slightly increased MMP-1, −9, and −13 expression, but not MMP-3 expression (Fig. 2A). Furthermore, treatment of cells with HGF increased MMP-2 mRNA expression dose-dependently (Fig. 2B); HGF also increased protein expression of MMP-2, as shown by Western blot analysis (Fig. 2C). MMP-2 expression was also increased in the cell culture supernatant, and its enzyme activity was up-regulated (Fig. 2C). We then directly confirmed whether MMP-2 is involved in HGF-induced cell migration. JJ012 cells were transfected with MMP-2 or control siRNA for 24 h, and western blot analysis showed that the MMP-2 protein expression levels was suppressed by transfection with *MMP2* siRNA (Fig. 2D). In addition, transfection of cells with *MMP2* siRNA or pretreatment of cells with MMP-2 inhibitor reduced HGF-induced cell migration (Fig. 2D).

We also established cells expressing HGF-shRNA to confirm whether HGF mediated cell migration and MMP-2 expression in human chondrosarcoma cells. The protein expression of HGF was markedly inhibited by HGF-shRNA orientation in JJ012/HGF-shRNA cells (Fig. 2E). Furthermore, knockdown of HGF also reduced MMP-2 expression in JJ012 cells (Fig. 2E). However, we did not find any difference in cell growth among these cells (data not shown). In contrast, knockdown of HGF expression inhibited the migratory ability by approximately 60% in JJ012 cells (Fig. 2F). Therefore, HGF promoted migration of human chondrosarcoma cells through up-regulation of MMP-2.

### HGF-directed Chondrosarcoma Cell Migration via the c-Met Receptor

**Figure 3:**
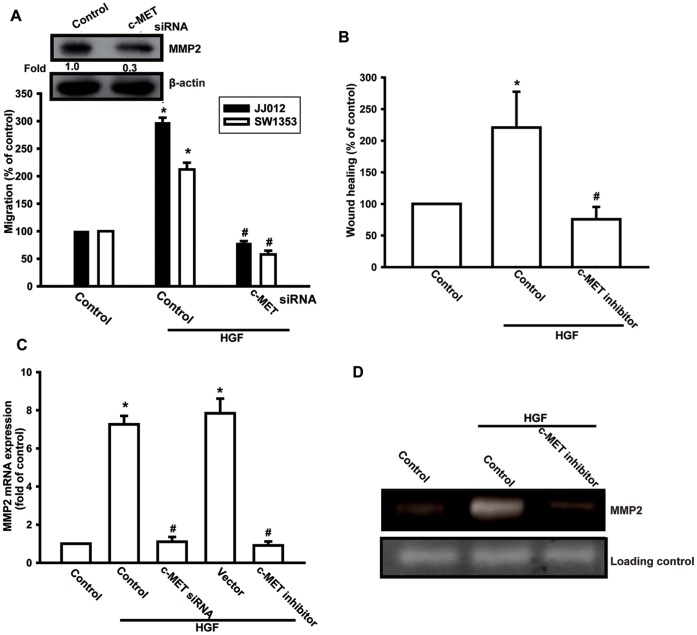
c-Met receptor is involved in HGF-mediated migration in human chondrosarcoma cells. (A–D) Cells were pretreated with the c-Met inhibitor (3 µM) for 30 min or transfected with c-Met siRNA for 24 h, followed by treatment with HGF for 24 h; cell migration and MMP-2 expression were then examined by Transwell, wound healing, qPCR, and zymography assays. Results are expressed as mean ± S.E. *p<0.05 compared with control; #*p*<0.05 compared with HGF-treated group.

It has been reported that HGF exerts its effects through activation of its specific receptor, c-Met [30]. We next examined whether c-Met receptor mediated HGF-promoted cell migration and MMP-2 expression. Transfection of cells with *c-met* siRNA or pretreatment of cells with c-Met inhibitor reduced HGF-increased cell migration (Figs. 3A and B). Furthermore, transfection or pretreatment with c-Met inhibitor or siRNA also reduced HGF-induced MMP-2 expression (Figs. 3C and D). Therefore, an interaction between HGF and c-Met appears to be very important for cell migration and MMP-2 expression in human chondrosarcoma.

### PI3K, Akt, and PKCβ Signaling Pathways are Involved in the HGF-mediated Cell Migration of Chondrosarcoma Cells

**Figure 4:**
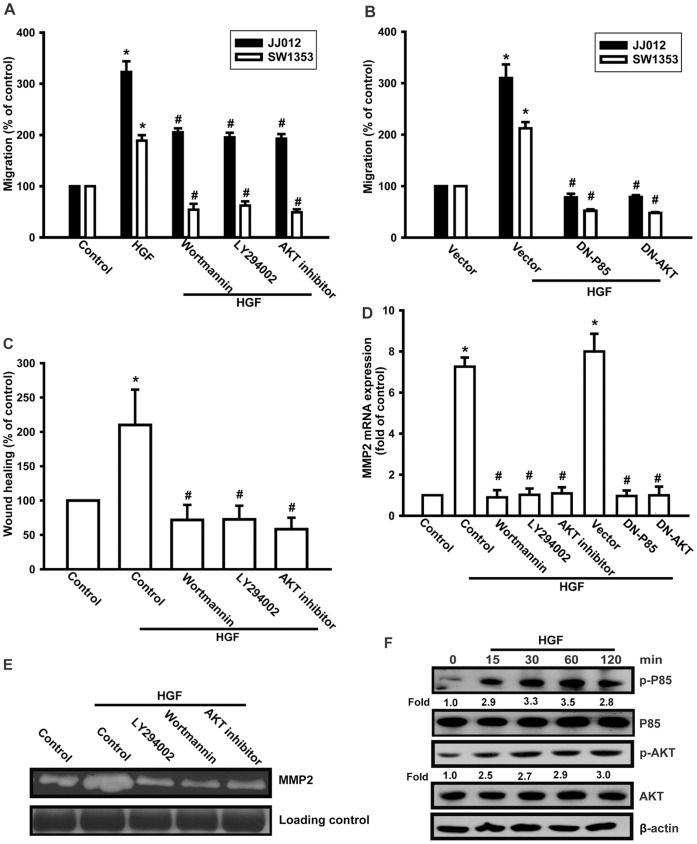
Involvement of PI3K/Akt signaling pathway in response to HGF in chondrosarcoma cells. (A–E) Cells were pretreated for 30 min with Ly294002 (10 µM), wortmannin (1 µM), and Akt inhibitor (1 µM) or transfected with dominant negative (DN) mutants of p85 and Akt for 24 h, followed by stimulation with HGF; cell migration and MMP-2 expression were then examined by Transwell, wound healing, qPCR, and zymography assays. (F) JJ012 cells were incubated with HGF for the indicated time intervals, and p-p85 and p-Akt expression was determined by western blotting. Results are expressed as mean ± S.E. *p<0.05 compared with control; #*p*<0.05 compared with HGF-treated group.

PI3K/Akt is a common downstream molecule of growth factor stimulation [31], [32], [33]. To examine whether activation of PI3K/Akt mediates HGF-triggered cell motility, chondrosarcoma cells were pretreated with PI3K inhibitors Ly294002 and wortmannin, or Akt inhibitor, for 30 min and were then incubated with HGF for 24 h. Pretreatment with PI3K (Ly294002 and wortmannin) or Akt inhibitor abolished HGF-induced cell migration and MMP-2 expression (Fig. 4A–E). In addition, transfection of cells with p85 or Akt mutants also blocked HGF-induced cell migration and MMP-2 expression (Figs. 4B and D). We then directly measured phosphorylation of p85 and Akt after HGF stimulation. Incubation of JJ012 cells led to a significant increase in phosphorylation of PI3K and Akt (Fig. 4F). Taken together, these results indicate that the PI3K and Akt pathways are involved in HGF-induced cell motility and MMP-2 expression.

**Figure 5:**
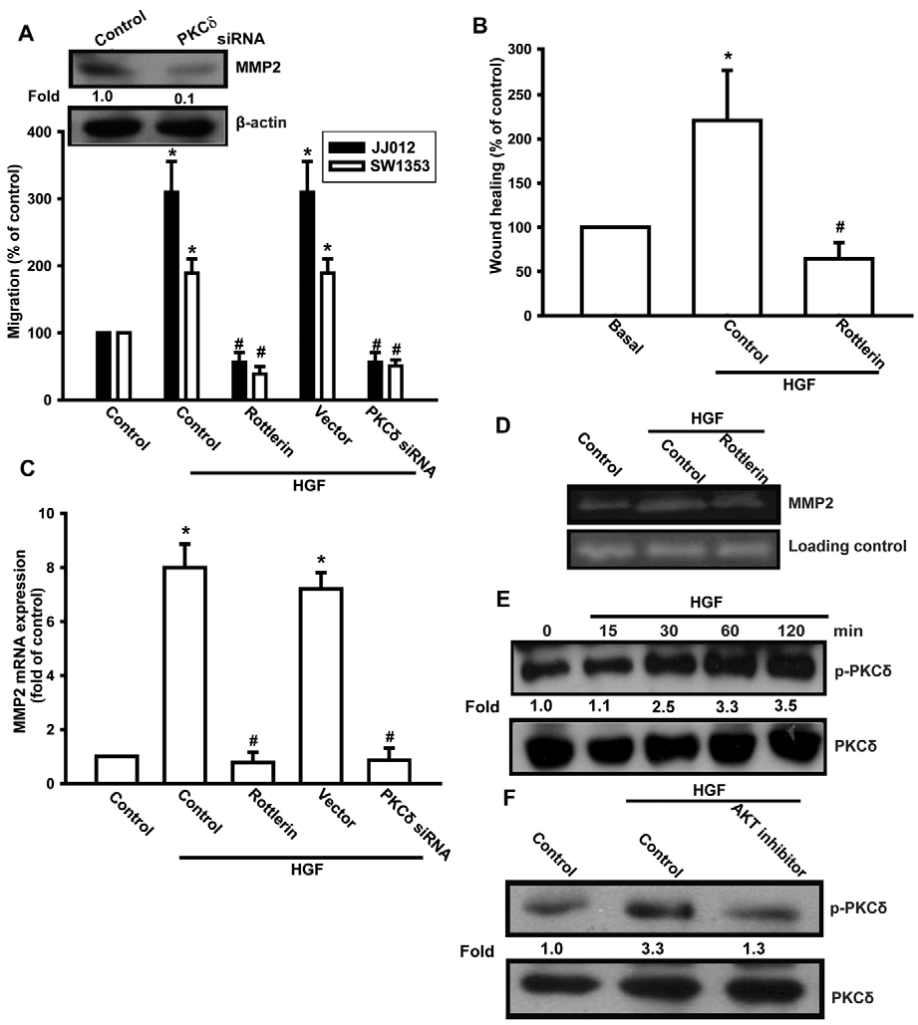
The PKCδ pathway is involved in HGF-mediated migration in human chondrosarcoma cells. (A–D) Cells were pretreated for 30 min with rottlerin (3 µM) or transfected with PKCδ siRNA 24 h, followed by stimulation with HGF; cell migration and MMP-2 expression were then examined by Transwell, wound healing, qPCR, and zymography assays. (E) JJ012 cells were incubated with HGF for the indicated time intervals, and p-PKCδ expression was determined by western blotting. (F) JJ012 cells were pretreated for 30 min with Akt inhibitor, followed by treatment with HGF for 30 min; p-PKCδ expression was examined by western blotting. Results are expressed as mean ± S.E. *p<0.05 compared with control; #*p*<0.05 compared with HGF-treated group.

PI3K/Akt-dependent PKCδ activation has been reported to regulate MMP expression [34]. We therefore investigated the effect of PKCδ in mediating HGF-induced cell migration and MMP-2 expression with the specific PKCδ inhibitor, rottlerin. HGF-induced cell migration and MMP-2 up-regulation was markedly attenuated by pretreatment of cells with rottlerin for 30 min or treatment of transfected cells with PKCδ siRNA for 24 h (Figs. 5A–C). Directly applied HGF also enhanced PKCδ phosphorylation in a time-dependent manner (Fig. 5E). In addition, HGF-induced PKCδ phosphorylation was inhibited upon pretreatment of cells with Akt inhibitor (Fig. 5F). Based on these results, HGF appears to act through a signaling pathway, involving PI3K, Akt, and PKCδ to enhance cell motility and MMP-2 expression in human chondrosarcoma.

### Involvement of NF-κB in HGF-induced Cell Migration and MMP-2 Expression

**Figure 6:**
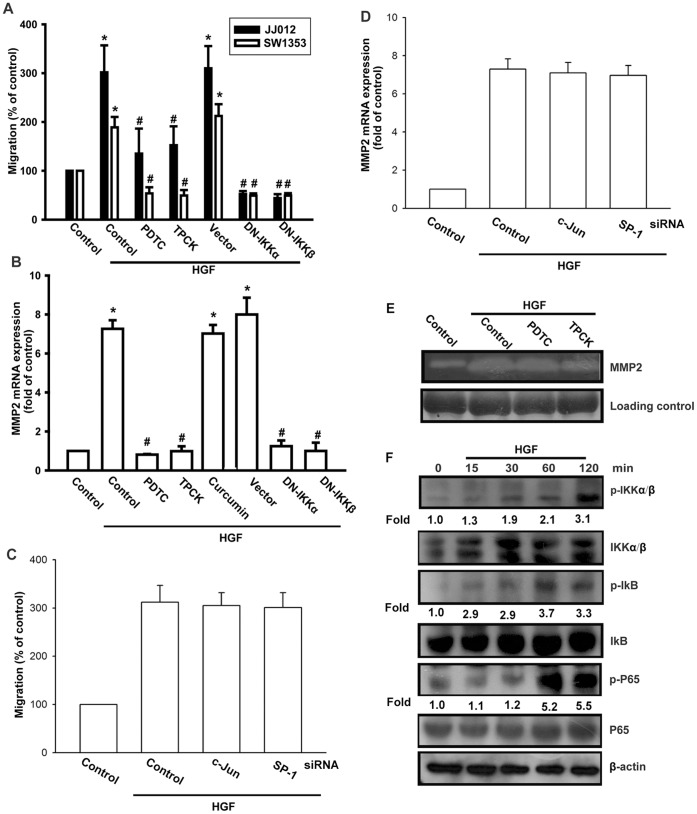
HGF induces cells migration and MMP-2 up-regulation through NF-κB. (A, B, and E) Cells were pretreated for 30 min with PDTC (10 µM), TPCK (3 µM), or curcumin (3 µM) or transfected with dominant-negative (DN) mutants of IKKα or IKKβ for 24 h, followed by stimulation with HGF. Cell migration and MMP-2 expression was examined by Transwell, qPCR, and zymography assays. (C and D) JJ012 cells were transfected with c-Jun or SP-1 siRNA followed by stimulation with HGF; cell migration and MMP-2 expression was examined by Transwell and qPCR assays. (F) JJ012 cells were incubated with HGF for the indicated time intervals, and p-IKK, p-IκB, and p-p65 expression was determined by western blotting. Results are expressed as mean ± S.E. *p<0.05 compared with control; #*p*<0.05 compared with HGF-treated group.

Recently, a study has documented that NF-κB activation is necessary for migration and invasion of human chondrosarcoma [35]. To examine whether NF-κB activation is involved in HGF-induced cell motility, the NF-κB inhibitor, PDTC, was used. We found that PDTC reduced the enhancement of cell motility and MMP-2 expression induced by HGF (Figs. 6A, B, and E). In addition, pretreatment of cells with an IκB protease inhibitor, TPCK, abolished the potentiating action on cell migration and MMP-2 expression (Figs. 6A, B, and E). The MMP-2 promoter also contains NF-κB, AP-1, and SP-1 binding sites. To further examine whether AP-1 and SP-1 activation are involved in HGF-mediated cell migration and MMP-2 expression, c-Jun and SP-1 siRNA were used. Transfection of cells with c-Jun or SP-1 siRNA did not affect HGF-mediated cell migration and MMP-2 expression (Figs. 6C and D). In addition, pretreatment of cells with AP-1 inhibitor (curcumin [36]) did not affect HGF-induced MMP-2 expression (Fig. 6B). Therefore, AP-1 and SP-1 are not involved in HGF-induced migration and MMP-2 expression. We next examined the upstream molecules involved in HGF-induced NF-κB activation. Transfection with IKKα or IKKβ mutants markedly inhibited HGF-induced cell migration and MMP-2 expression (Figs. 6A and B). On the other hand, incubation of cells with HGF induced IKKα/β phosphorylation in a time-dependent manner (Fig. 6F). These data suggest that IKKα/β activation is involved in HGF-induced MMP-2 expression and migration of human chondrosarcoma cells.

**Figure 7:**
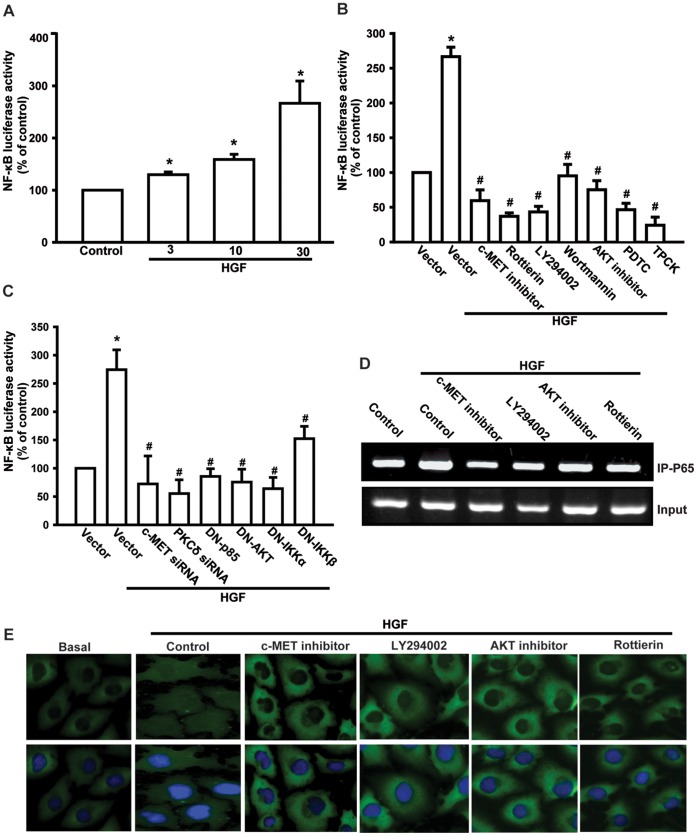
The PI3K/Akt/PKCδ pathway is involved in HGF-mediated NF-κB activation and MMP-2 expression. JJ012 cells were incubated with HGF for 24 h (A) or pretreated with c-Met inhibitor, Ly294002, wortmannin, Akt inhibitor, rottlerin, PDTC, and TPCK for 30 min (B) or were transfected with c-Met siRNA, PKCδ siRNA, p85 mutant, Akt mutant, IKKα mutant, and IKKβmutant (C) before exposure to HGF. NF-κB luciferase activity was measured, and the results were normalized to the β-galactosidase activity and expressed as mean ± S.E.M. for 3 independent experiments, each performed in triplicate. (D) JJ012 cells were pretreated with c-Met inhibitor, Ly294002, wortmannin, Akt inhibitor, or rottlerin, then stimulated with HGF for 120 min, before a chromatin immunoprecipitation assay was performed. Chromatin was immunoprecipitated with anti-p-65. One percentage of the precipitated chromatin was assayed to verify equal loading (input). (E) Upper panel, p65 staining; lower panel, p65 staining merged with nucleus staining. JJ012 cells were pretreated with c-Met inhibitor, Ly294002, wortmannin, Akt inhibitor, or rottlerin, and then stimulated with HGF for 120 min, and p65 immunofluorescence staining then examined. Results are expressed as mean ± S.E. *p<0.05 compared with control; #*p*<0.05 compared with HGF-treated group.

The role of NF-κB activation was further evaluated by analyzing NF-κB luciferase activity and p65 translocation into nucleus, as well as by a chromatin immunoprecipitation assay. Treatment of JJ012 cells with HGF caused phosphorylation of IκBα and p65 in a time-dependent manner (Fig. 6F). Furthermore, stimulation of JJ012 cells for 24 h enhanced κB-luciferase activity dose-dependently (Fig. 7A). HGF-mediated κB-luciferase activity was also inhibited by treatment with the c-Met inhibitor, Ly294002, wortmannin, Akt inhibitor, rottlerin, PDTC, and TPCK (Fig. 7B). On the other hand, co-transfection with p85, Akt, IKKα and IKKβ mutants or c-Met and PKCδ siRNAs also abolished HGF-increased NF-κB luciferase activity (Fig. 7C). We then investigated whether p65 binds to the NF-κB element on the MMP-2 promoter after HGF stimulation. *In vivo* recruitment of p65 to the MMP-2 promoter (−673 to −517) was assessed using a chromatin immunoprecipitation assay [37]. HGF stimulation promoted p65 binding to the NF-κB element of the MMP-2 promoter (Fig. 7D). This was attenuated by the c-Met inhibitor, Ly294002, wortmannin, Akt inhibitor, and rottlerin (Fig. 7D). In addition, c-Met inhibitor, Ly294002, wortmannin, Akt inhibitor, and rottlerin also reduced HGF-mediated p65 translocation into the nucleus (Fig. 7E, upper panel, p65 staining; lower panel, p65 staining merged with nucleus staining). Taken together, these data suggest that activation of the c-Met receptor, PI3K, Akt, and PKCδ are required for HGF-induced NF-κB activation in human chondrosarcoma cells.

## Discussion

The metastatic potential of conventional chondrosarcomas correlates well with the histologic grade of the tumor. But due to the relatively indolent growth rates of many low- and moderate-grade chondrosarcomas, approximately 15% of patients dying from metastatic disease do so >5 years after their initial diagnosis [38]. Therefore, it is important to develop effective adjuvant therapy for preventing chondrosarcoma metastases. In this study, we hypothesized that HGF may help to direct the metastasis of chondrosarcoma cells. Using immunohistochemistry, we showed that the protein expression levels of HGF in chondrosarcoma patients were significantly higher than those in normal cartilage. We also provided evidence that the mRNA level of HGF in chondrosarcoma patients were higher than in normal cartilage and normal bone. Knockdown of HGF using HGF shRNA reduced cell motility by approximately 60% in JJ012 cells. Our data suggested that expression of HGF is associated with a metastatic phenotype of chondrosarcoma cells. Moreover, directly-administered exogenous HGF promoted cell migration, invasion, and wound healing activity in chondrosarcoma cells. One of the mechanisms underlying HGF-directed migration was transcriptional up-regulation of MMP-2 and activation of the c-Met receptor, PI3K, Akt, PKCδ, and NF-κB pathways.

c-Met receptor on the cell surface is responsible for HGF-mediated cell motility [39]. Here, we confirmed that c-Met receptor is required for HGF-induced cell motility and MMP-2 expression. Pretreatment of cells with c-Met inhibitor reduced HGF-induced cell migration and MMP-2 expression. This was further confirmed by the finding that c-Met siRNA treatment inhibited the HGF-mediated enhancement of cell migration and MMP-2 production. Here, we also showed that the interaction between HGF and c-Met is very important for MMP-2 up-regulation and cell motility of human chondrosarcoma cells.

ECM degradation is an essential step in cancer migration and invasion. MMP-1, −2, −3, −9, and −13 have been found to regulate metastasis of human chondrosarcoma [14], [40]. On the other hand, activation of MMPs is also involved in HGF-mediated cell motility in human cancer cells [41]. In this study, we found that HGF induced MMP-2 expression and secretion in human chondrosarcoma cells. In addition, inhibition of HGF-enhanced MMP-2 protein expression with siRNA significantly suppressed HGF-induced migration. Therefore, MMP-2 may be the HGF-responsive mediator, and its degradation of ECM may lead to subsequent cancer migration and metastasis. To further confirm that the c-Met receptor, PI3K, Akt, PKCδ, and NF-κB are required for HGF-induced cell motility and MMP-2 expression, MMP-2 knockdown was rescued with transfection of an MMP-2 cDNA expression vector. Transfection of cells with MMP-2 cDNA rescued the effect of MMP-2, c-Met, PI3K, Akt, PKCδ, and NF-κB inhibitors in HGF-mediated cell motility (Figure S1). These data provided evidence that HGF induced cell migration and MMP-2 up-regulation through the c-Met, PI3K, Akt, PKCδ, and NF-κB pathways.

There are several binding sites for a number of transcription factors, including NF-κB, AP-1, and SP-1, in the 5′-region of *MMP2* [42]. However, we found that NF-κB inhibitors, but not c-Jun and SP-1 siRNA, reduced HGF-increased cell motility and MMP-2 expression. Therefore, the NF-κB binding site is likely to be an important site for HGF-induced MMP-2 expression and cell migration in human chondrosarcoma. However, we did not examine the role of other transcription factors, including CREB and p53, which bind to the *MMP2* promoter region. Whether other transcription factors are involved in HGF-induced MMP-2 expression in chondrosarcoma needs further examination.

PKCδ has been reported to be the downstream effector of PI3K/Akt [43]. We demonstrated that rottlerin (a specific PKCδ inhibitor) inhibited HGF-induced migration and MMP-2 expression of cells, suggesting that PKCδ activation is a requisite event in HGF-induced cell motility in these cells. This was confirmed by the observation that PKCδ siRNA inhibited the enhancement of migration and MMP-2 expression in human chondrosarcoma cells. Incubation of JJ012 cells with HGF increased PKCδ phosphorylation. Pretreatment of cells with Akt inhibitor reduced HGF-mediated PKCδ phosphorylation. These data suggested that the PI3K/Akt-dependent PKCδ pathway is required for HGF-induced MMP-2 expression and cancer migration. Importantly, this study presented the first evidence that the PI3K/Akt/PKCδ pathway is involved in cancer metastasis. Therefore, this pathway is inactive during the normal biological state, but becomes active in the pathological state. Moreover, we have provided evidence that the PI3K/Akt/PKCδ pathway mediated HGF-induced cell metastasis. Further investigation will be needed to determine whether this pathway is a common pathway for cancer migration and metastasis.

The prognosis of patients with chondrosarcoma distant metastases is generally considered very poor; hence, preventing human chondrosarcoma metastasis is important. Our study demonstrated that HGF increases the activity of MMP-2 via c-Met receptor-, PI3K-, Akt-, PKCδ-, IKKα/β-, and NF-κB-dependent pathways and enhanced the migration of human chondrosarcoma cells. The elucidation of the HGF-mediated signaling pathway sheds light on the mechanism underlying human chondrosarcoma metastasis and may lead to the development of effective therapy in future.

## Supporting Information

Figure S1 (DOC)
