# Expression of Concern: HGF and c-Met Interaction Promotes Migration in Human Chondrosarcoma Cells

**DOI:** 10.1371/journal.pone.0297300

**Published:** 2024-01-11

**Authors:** The PLOS ONE Editors

After this article [1] was published, concerns were raised about results presented in Figs 2, 3, 5 and 6. Specifically:

- The MMP2 panel in Fig 2D appears similar to the MMP2 panel in Fig 2E.
- The β-actin panel in Fig 3A is duplicated as the β-actin panel in Fig 5A.
- The β-actin panel in Fig 1C in [2] is duplicated as the β-actin panel in Fig 6F in [1].

In response to queries about the experiments in Fig 2, the corresponding author stated that the Fig 2D MMP2 and Fig 2E MMP2 panels appear similar but are from different blots. Higher resolution blots for the Fig 2D MMP2 and Fig 2E MMP2 panels and repeat blots for these panels from the time of the original experiments and later experiments are provided here in S1–S3 Files. The *PLOS ONE* Editors remain concerned that the cropped underlying data provided for the Fig 2 MMP2 panels appear more similar than would be expected from independent results.

The authors stated that the panels of concern in Figs 3A, 5A and 6F are incorrect in [1], and that the MMP2 panels in Figs 3A and 5A are likewise incorrect. They provided updated versions of Figs 3, 5 and 6 in which the panels of concern have been replaced. According to the corresponding author, the replacement panels report the correct data from the original experiments.

The corresponding author stated that the underlying uncropped and unadjusted blots for all published, corrected and repeat blots are not available.

Higher resolution versions of the corrected panels and cropped images of blots from later repeat experiments are provided here in S4–S8 Files. Additional data provided in support of this study are in S9 File.

In light of the unresolved Fig 2 concern, the extent of image issues in this article, and the unavailability of the original uncropped image data for the experiments of concern, the *PLOS ONE* Editors issue this Expression of Concern.

**Fig 3:**
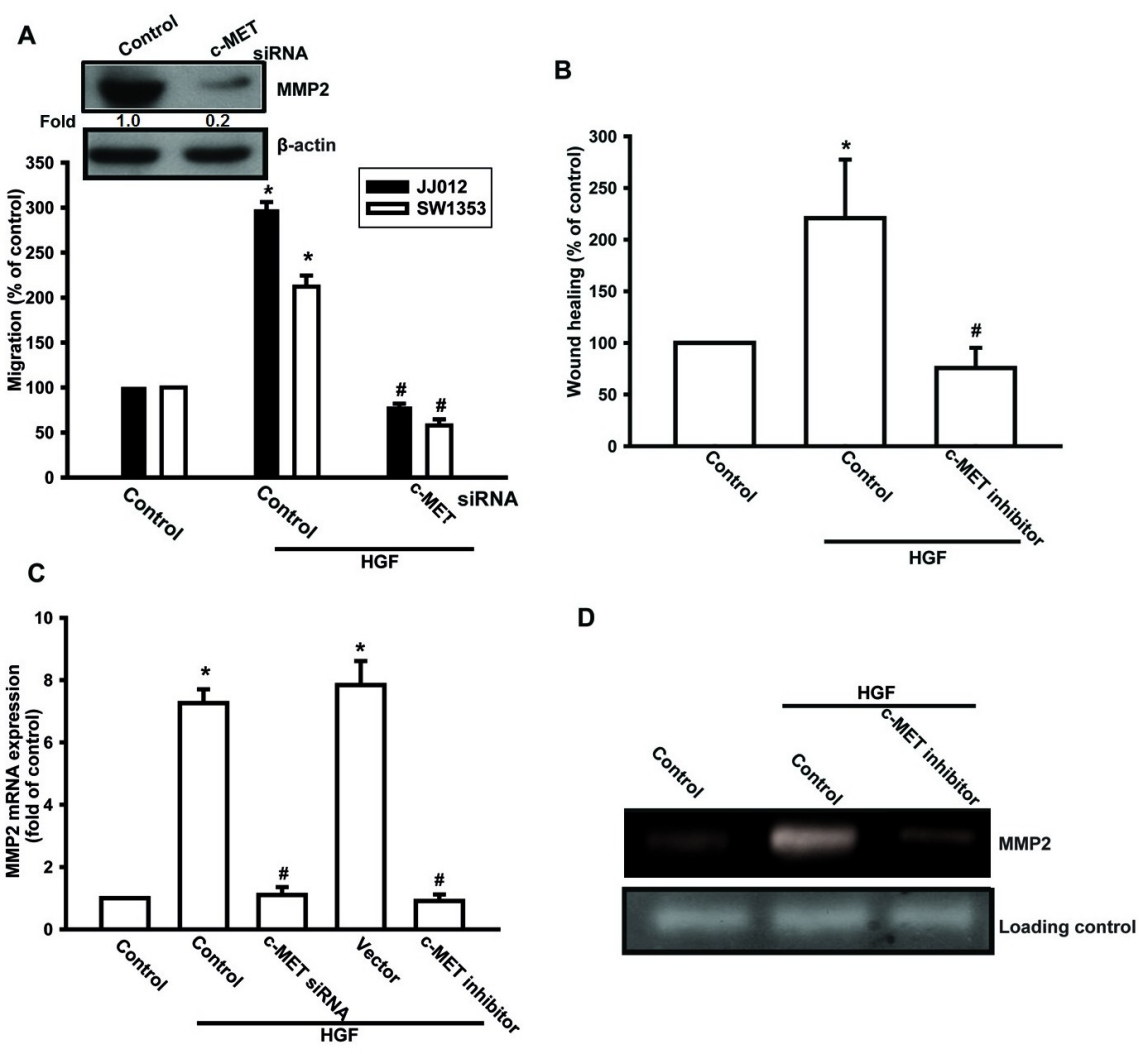
c-Met receptor is involved in HGF-mediated migration in human chondrosarcoma cells. (A–D) Cells were pretreated with the cMet inhibitor (3 μM) for 30 min or transfected with c-Met siRNA for 24 h, followed by treatment with HGF for 24 h; cell migration and MMP-2 expression were then examined by Transwell, wound healing, qPCR, and zymography assays. Results are expressed as mean ± S.E. *p < 0.05 compared with control; #*p* < 0.05 compared with HGF-treated group.

**Fig 5:**
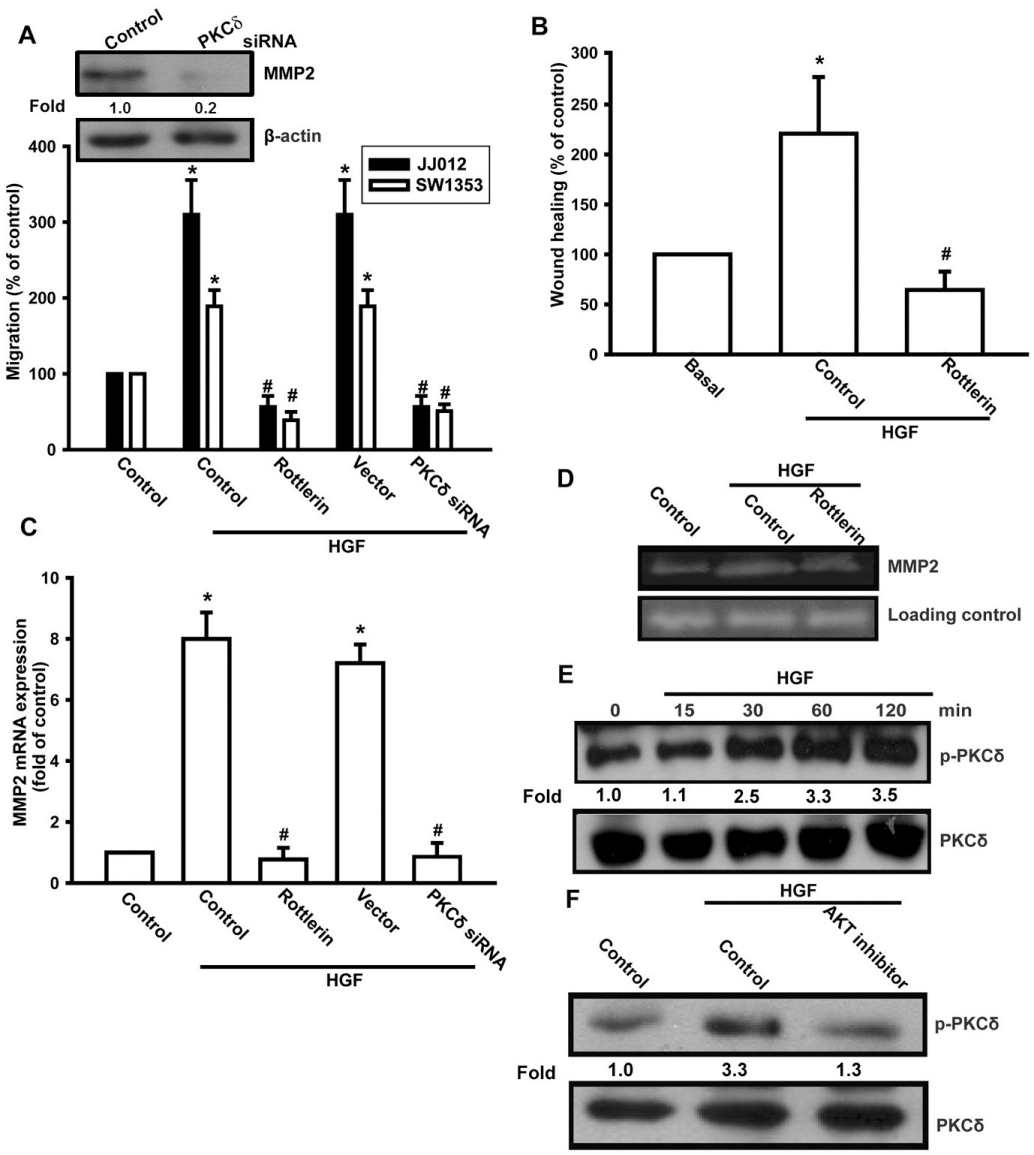
The PKCδ pathway is involved in HGF-mediated migration in human chondrosarcoma cells. (A–D) Cells were pretreated for 30 min with rottlerin (3 μM) or transfected with PKCδ siRNA 24 h, followed by stimulation with HGF; cell migration and MMP-2 expression were then examined by Transwell, wound healing, qPCR, and zymography assays. (E) JJ012 cells were incubated with HGF for the indicated time intervals, and pPKCδ expression was determined by western blotting. (F) JJ012 cells were pretreated for 30 min with Akt inhibitor, followed by treatment with HGF for 30 min; p-PKCδ expression was examined by western blotting. Results are expressed as mean ± S.E. *p < 0.05 compared with control; #*p* < 0.05 compared with HGF-treated group.

**Fig 6:**
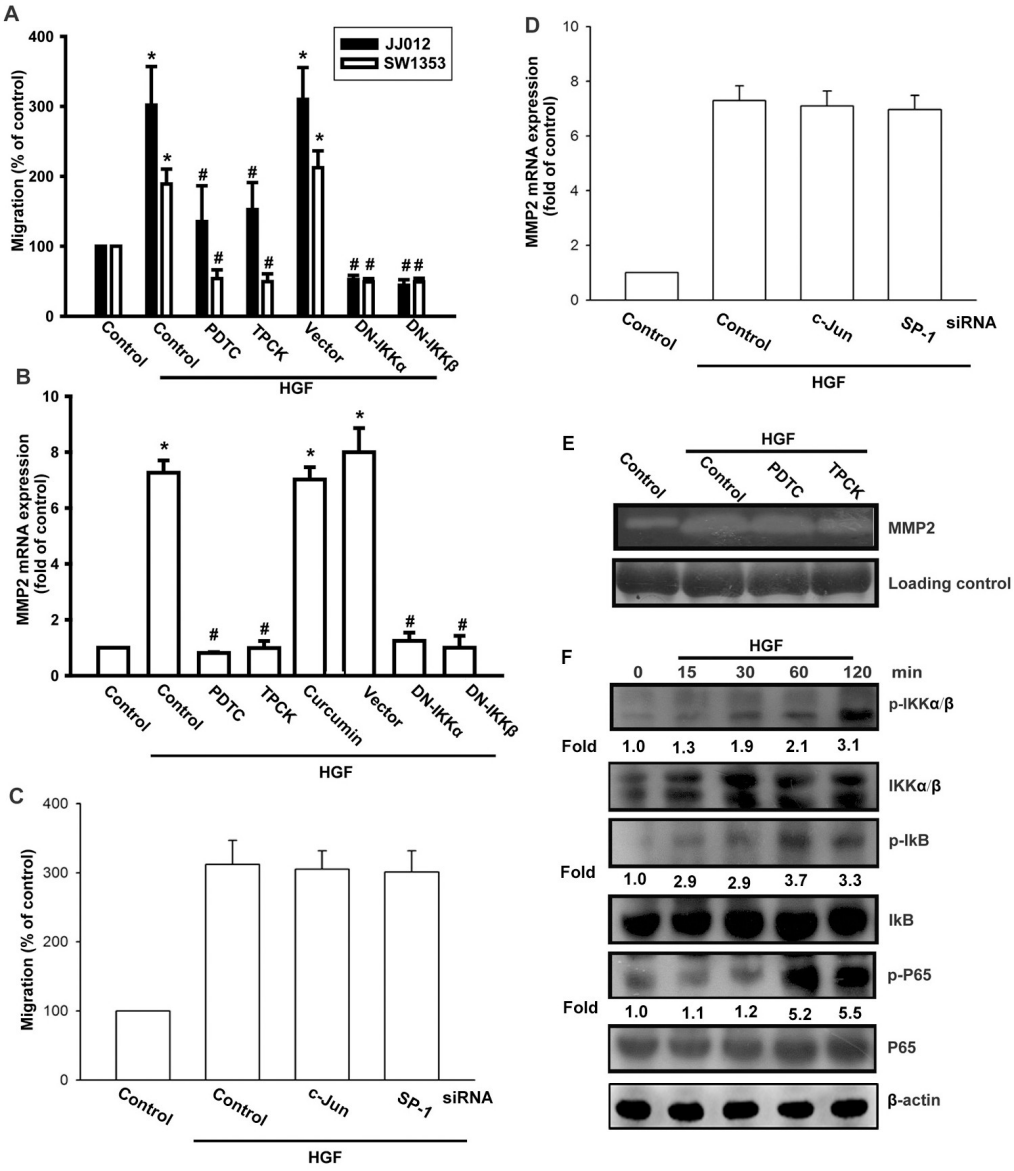
HGF induces cells migration and MMP-2 up-regulation through NF-κB. (A, B, and E) Cells were pretreated for 30 min with PDTC (10 μM), TPCK (3 μM), or curcumin (3 μM) or transfected with dominant-negative (DN) mutants of IKKα or IKKβ for 24 h, followed by stimulation with HGF. Cell migration and MMP-2 expression was examined by Transwell, qPCR, and zymography assays. (C and D) JJ012 cells were transfected with cJun or SP-1 siRNA followed by stimulation with HGF; cell migration and MMP-2 expression was examined by Transwell and qPCR assays. (F) JJ012 cells were incubated with HGF for the indicated time intervals, and p-IKK, p-IκB, and p-p65 expression was determined by western blotting. Results are expressed as mean ± S.E. *p < 0.05 compared with control; #*p* < 0.05 compared with HGF-treated group.

## Supporting information

S1 File Published and repeat blots for the Fig 2D MMP2 panel from the time of the original experiments. (JPG)

S2 File Published and repeat blots for the Fig 2E MMP2 panel from the time of the original experiments. (JPG)

S3 File Published and repeat blots for the Fig 2D MMP2 and Fig 2E MMP2 panels from the time of the original experiments and later experiments. (JPG)

S4 File Corrected and repeat blots for the Fig 3A β-actin panel from the time of the original experiments. (JPG)

S5 File Corrected and repeat blots for the Fig 5A β-actin panel from the time of the original experiments. (JPG)

S6 File Corrected and repeat blots for the Figs 3A and 5A β-actin and MMP2 panels from the time of the original experiments and later experiments. (JPG)

S7 File Corrected and repeat blots for the Fig 6F β-actin panel from the time of the original experiments and later experiments. (JPG)

S8 File Published and repeat blots for Fig 6F (except for the β-actin panel) from the time of the original experiments. (JPG)

S9 File Individual-level data for the charts in Figs 2, 3, 5 and 6 and repeat charts for these figures from later experiments. Published and repeat blots for Figs 2C, 3D, 5D–5F and 6E from the time of the original experiments.
(ZIP)
