# Supplementary material for: HGF and c-Met Interaction Promotes Migration in Human Chondrosarcoma Cells
Source: PLoS One. 2013 Jan 8;8(1):e53974. doi: 10.1371/journal.pone.0053974 (PMC3540013; doi:10.1371/journal.pone.0053974)
Supplement: Figure S1 — (DOC) [file pone.0053974.s001.doc]

**Supplementary** **Data**


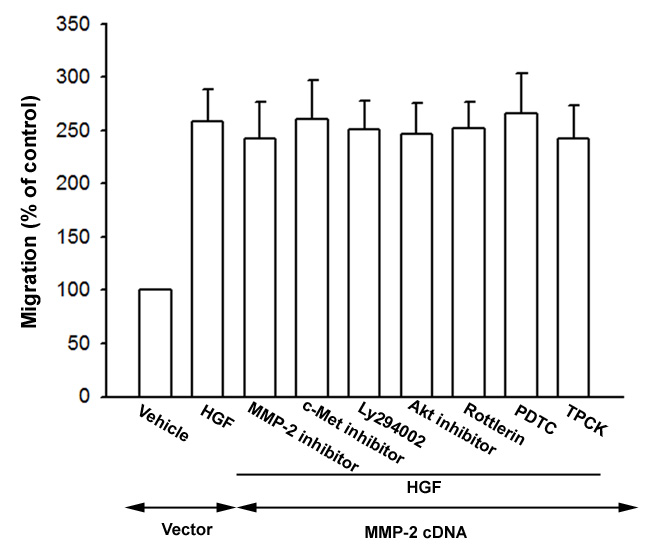


**Figure S1. MMP-2 cDNA rescues the MMP-2, c-Met, PI3K, Akt, PKCδ, and NF-κB inhibitor in HGF-mediated cell motility.** JJ012 cells were transfected with vector or MMP-2 cDNA for 24 h, and then pretreated for 30 min with MMP-2 inhibitor, c-Met inhibitor, Ly294002, Akt inhibitor, rottlerin, PDTC, and TPCK followed by stimulation with HGF, the cell migration was examined by Transwell. Results are expressed as the mean ± S.E.
