# Supplementary figures and images for: Expression of Concern: HGF and c-Met Interaction Promotes Migration in Human Chondrosarcoma Cells
Source: PLoS One. 2024 Jan 11;19(1):e0297300. doi: 10.1371/journal.pone.0297300 (PMC10783710; doi:10.1371/journal.pone.0297300)

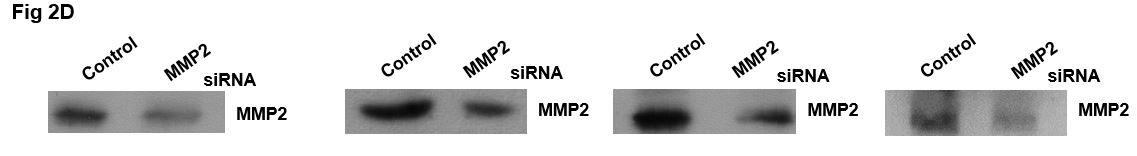

Supplement: S1 File — (JPG) [file pone.0297300.s001.jpg]

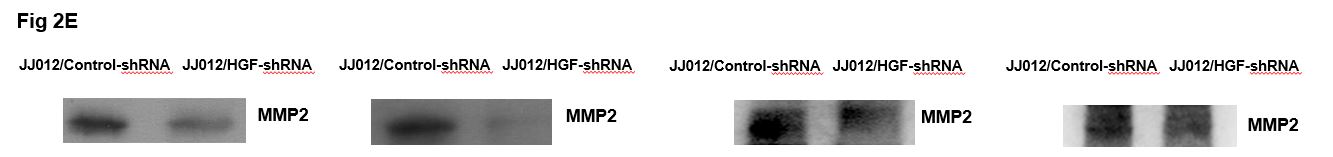

Supplement: S2 File — (JPG) [file pone.0297300.s002.jpg]

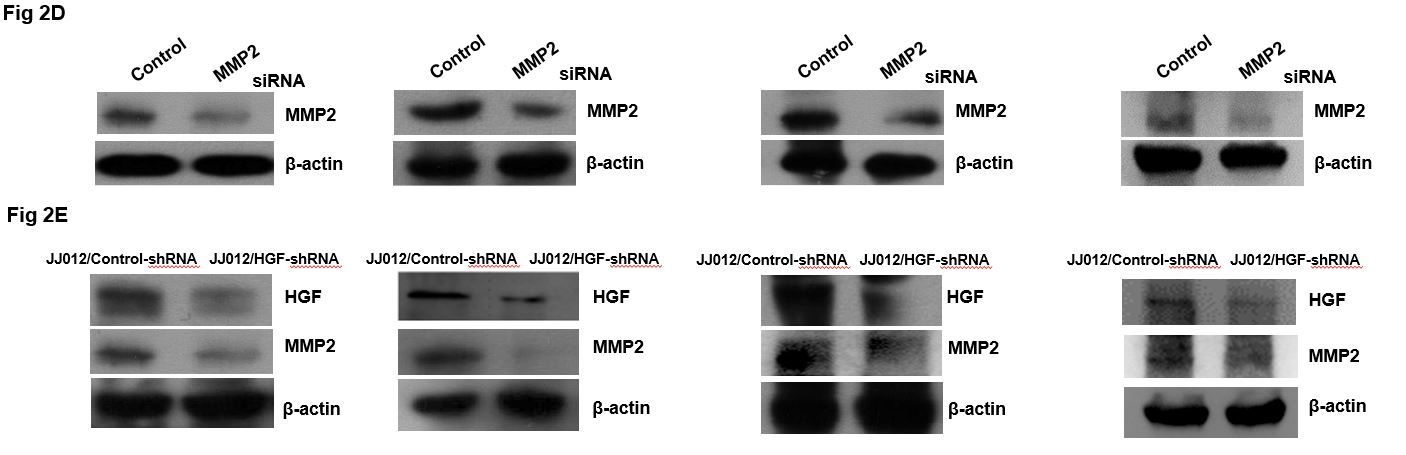

Supplement: S3 File — (JPG) [file pone.0297300.s003.jpg]

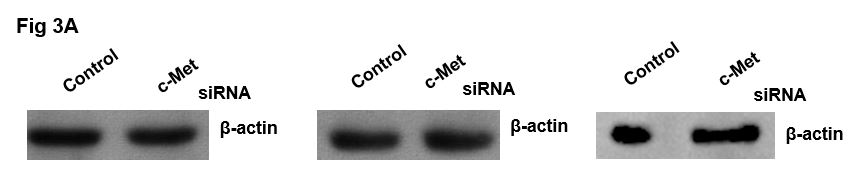

Supplement: S4 File — (JPG) [file pone.0297300.s004.jpg]

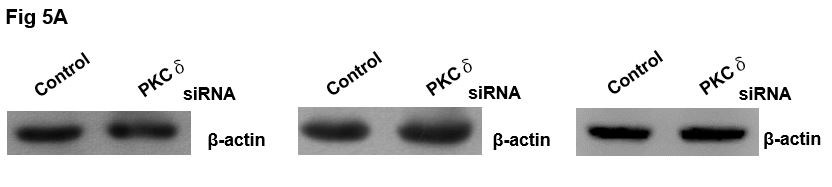

Supplement: S5 File — (JPG) [file pone.0297300.s005.jpg]

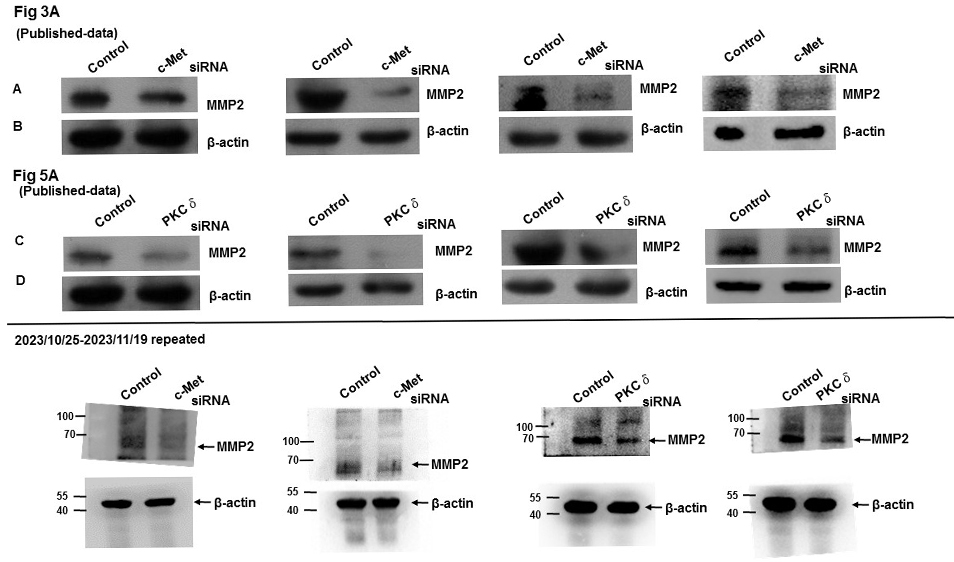

Supplement: S6 File — (JPG) [file pone.0297300.s006.jpg]

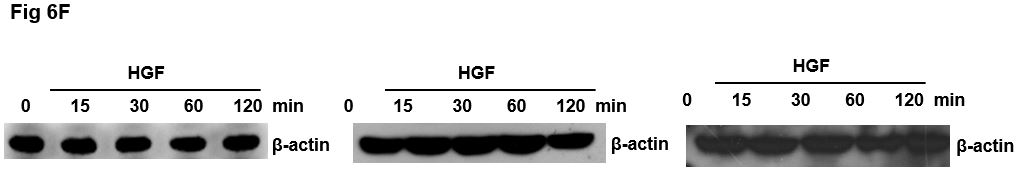

Supplement: S7 File — (JPG) [file pone.0297300.s007.jpg]

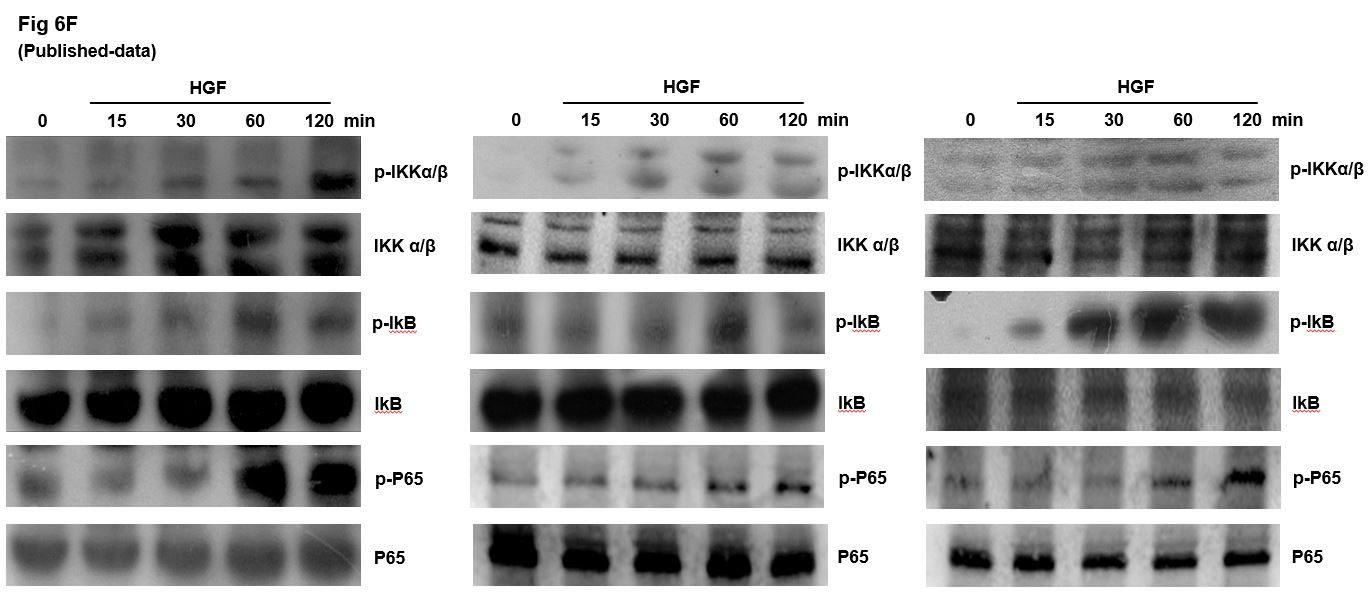

Supplement: S8 File — (JPG) [file pone.0297300.s008.jpg]

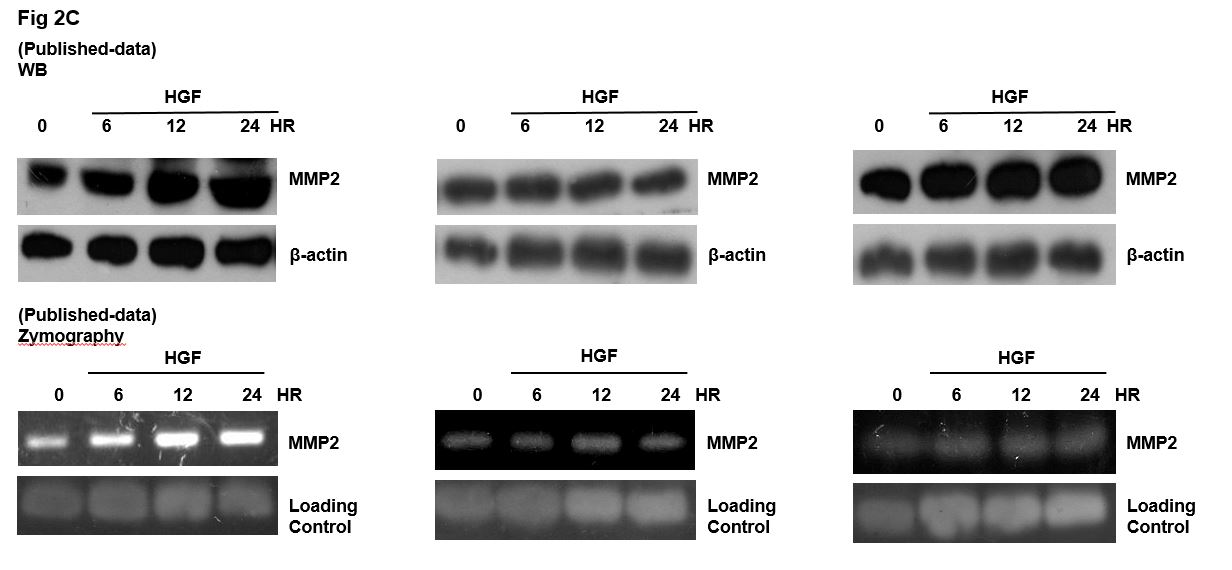

Supplement: S9 File — Published and repeat blots for Figs 2C, 3D, 5D–5F and 6E from the time of the original experiments. (ZIP) [file pone.0297300.s009.zip › Fig 2c_ MMP2 blot zymography.jpg]

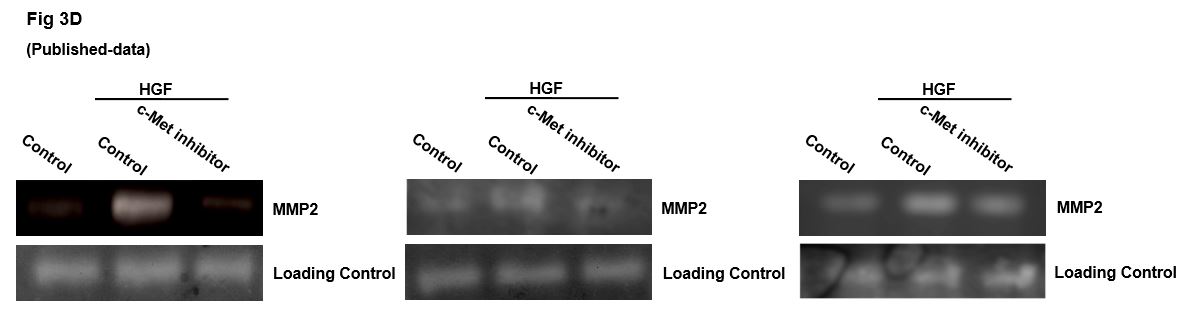

Supplement: S9 File — Published and repeat blots for Figs 2C, 3D, 5D–5F and 6E from the time of the original experiments. (ZIP) [file pone.0297300.s009.zip › Fig 3d_ MMP2 zymography.jpg]

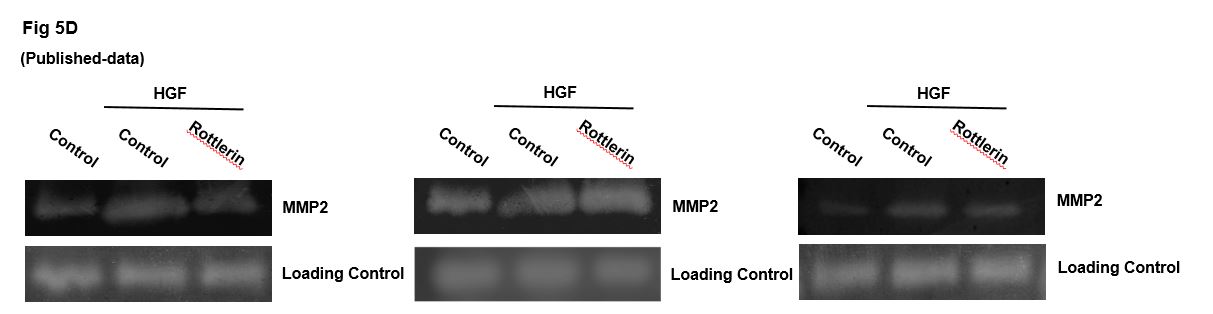

Supplement: S9 File — Published and repeat blots for Figs 2C, 3D, 5D–5F and 6E from the time of the original experiments. (ZIP) [file pone.0297300.s009.zip › Fig 5d_ MMP2 zymography.jpg]

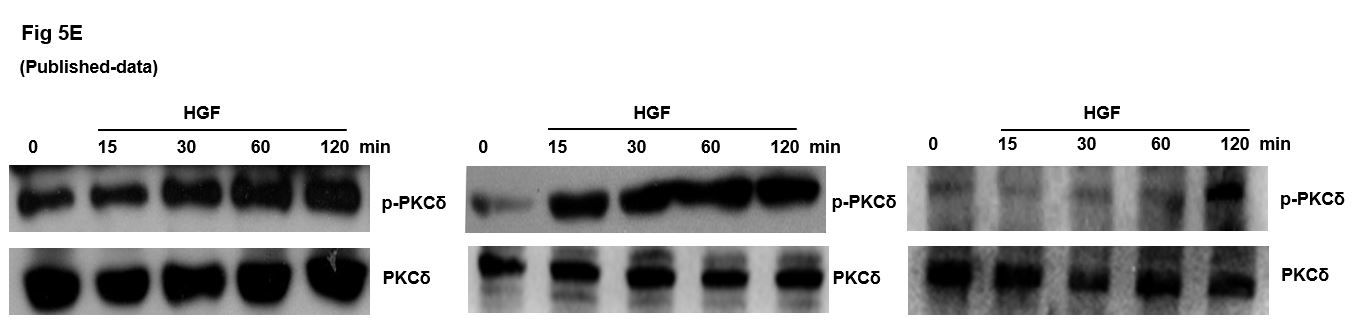

Supplement: S9 File — Published and repeat blots for Figs 2C, 3D, 5D–5F and 6E from the time of the original experiments. (ZIP) [file pone.0297300.s009.zip › Fig 5e_ p-PKC blot.jpg]

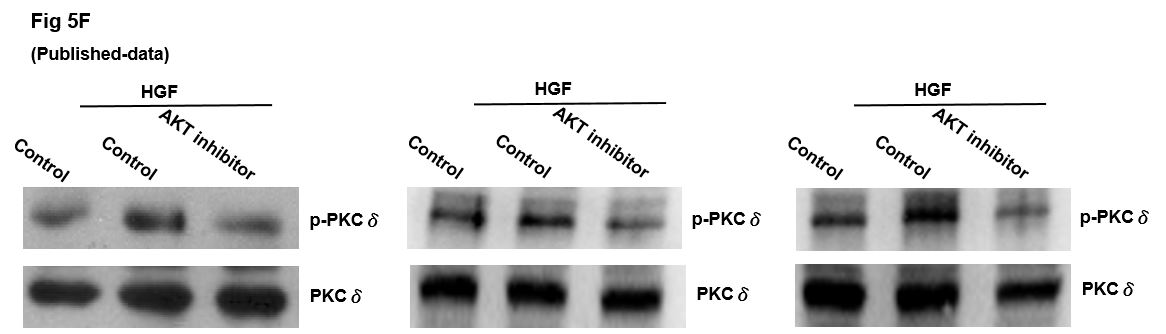

Supplement: S9 File — Published and repeat blots for Figs 2C, 3D, 5D–5F and 6E from the time of the original experiments. (ZIP) [file pone.0297300.s009.zip › Fig 5f_ p-PKC blot.jpg]

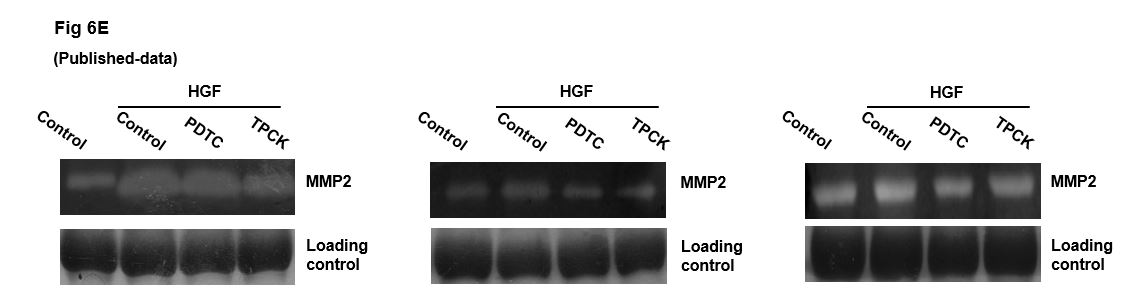

Supplement: S9 File — Published and repeat blots for Figs 2C, 3D, 5D–5F and 6E from the time of the original experiments. (ZIP) [file pone.0297300.s009.zip › Fig 6e_ MMP2 zymography.jpg]

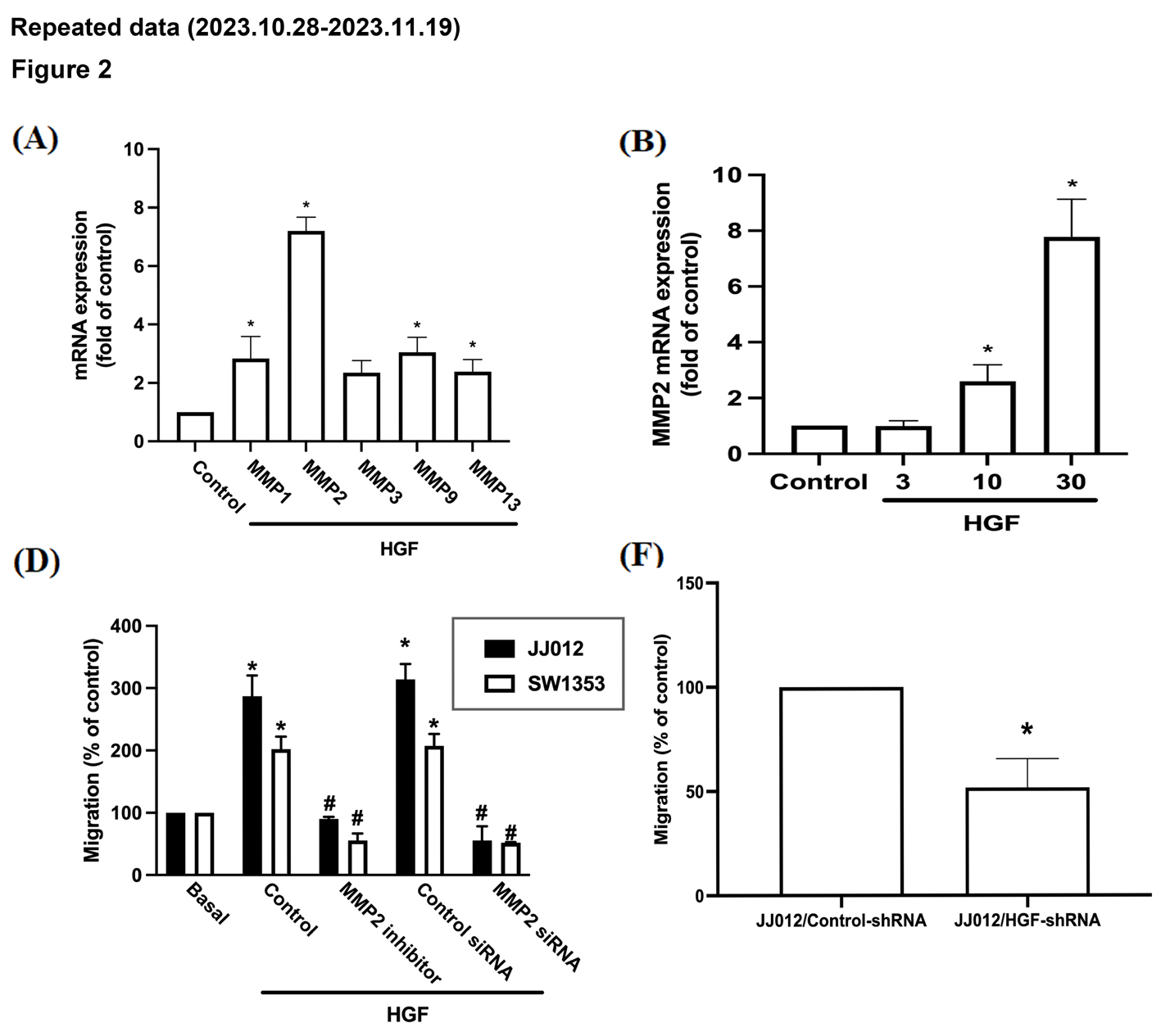

Supplement: S9 File — Published and repeat blots for Figs 2C, 3D, 5D–5F and 6E from the time of the original experiments. (ZIP) [file pone.0297300.s009.zip › Figure 2-repeated.jpg]

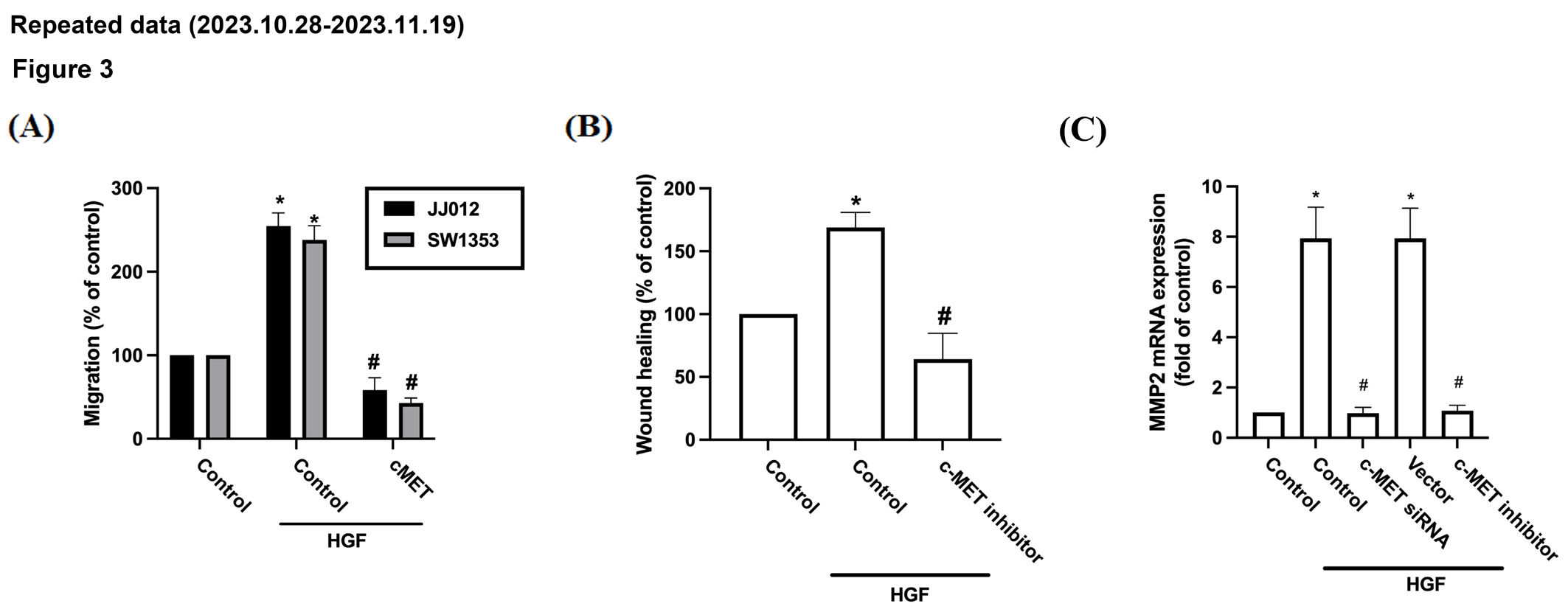

Supplement: S9 File — Published and repeat blots for Figs 2C, 3D, 5D–5F and 6E from the time of the original experiments. (ZIP) [file pone.0297300.s009.zip › Figure 3-repeated.jpg]

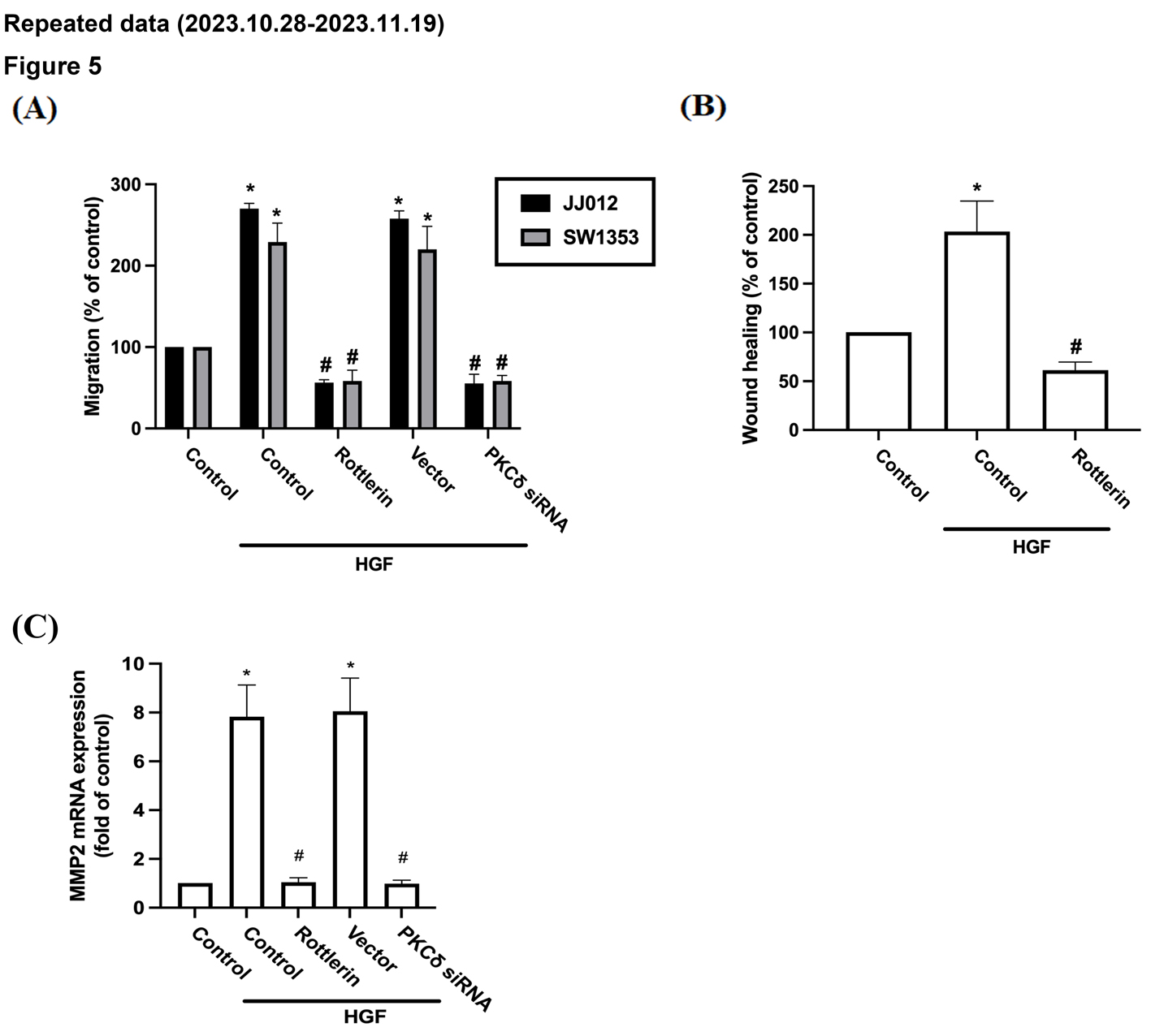

Supplement: S9 File — Published and repeat blots for Figs 2C, 3D, 5D–5F and 6E from the time of the original experiments. (ZIP) [file pone.0297300.s009.zip › Figure 5-repeated.jpg]

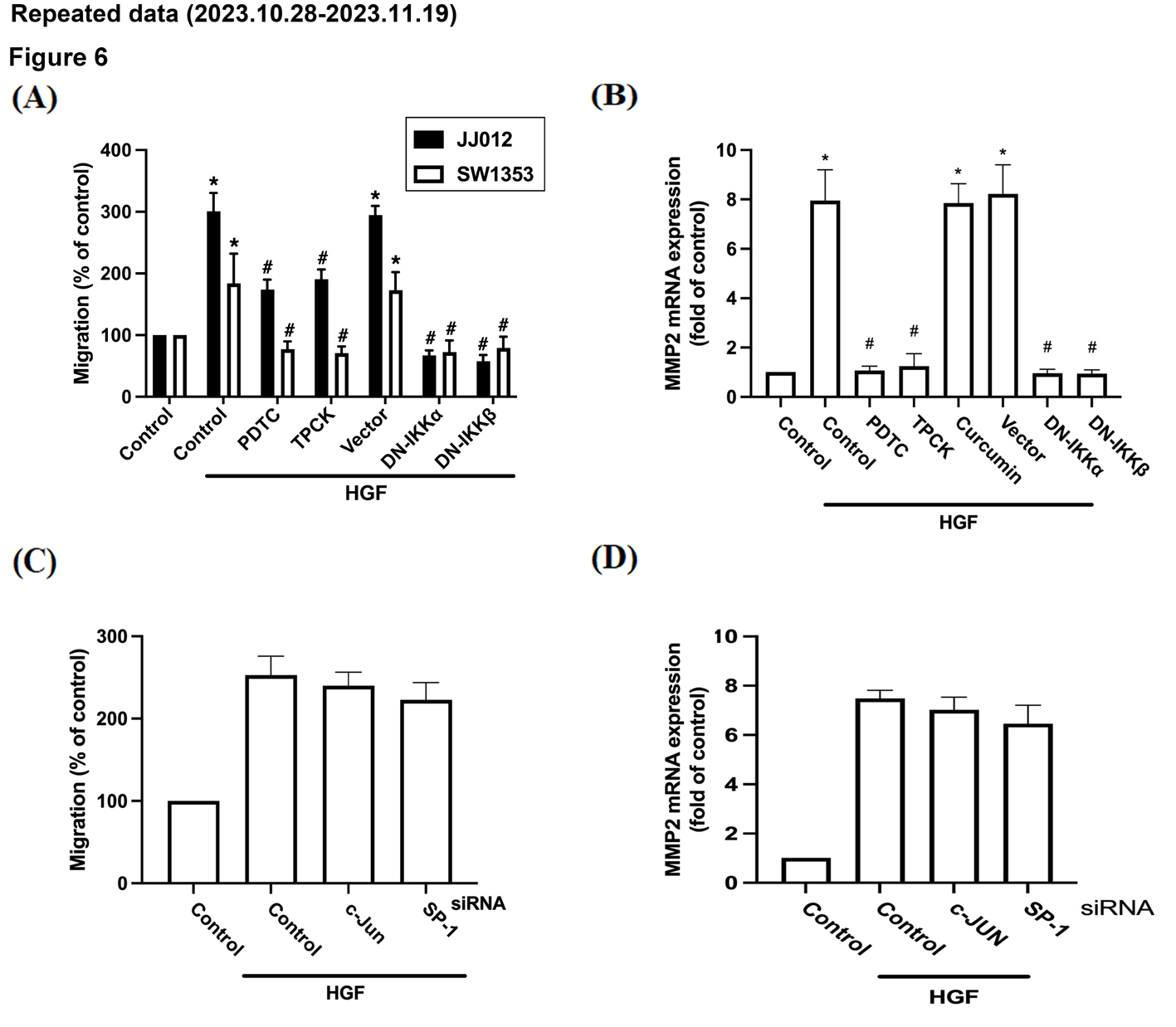

Supplement: S9 File — Published and repeat blots for Figs 2C, 3D, 5D–5F and 6E from the time of the original experiments. (ZIP) [file pone.0297300.s009.zip › Figure 6-repeated.jpg]
